# Letter from Philadelphia

**Published:** 1868-06-01

**Authors:** 


### *PHILADELPHIA CORRESPONDENCE.*

PHILADELPHIA, May 9, 1868.

*Chicago Medical Journal:*

R. J., aged 40, colored, *Epulis*, tumor inside of mouth, connected with lower jaw bone, situated in front. Two incisions were made, and the teeth of lower jaw in front removed. Lower lip was separated from the gum as far down as the alveolæ. The portion of bone to which the tumour was attached was now removed. Parts brought together with interrupted suture. Wash of carbolic acid gr.x to ℥i. water used as dressing. Nine days after patient presented herself cured.

J. P., aged 35. Apparent dislocation of the forearm backwards, with fracture of the external condyle. The limb was in an extended position, and of but little use to the patient. Three months since he had a fall and this is the result. Chloroform was administered. The olecranon was found to project considerably back of the posterior surface of the humerus. An incision one-fourth of an inch was made over the olecranon process, and a drill introduced. With this the olecranon was fractured, and the arm bent at an acute angle across the breast. The wound was closed with twisted suture and collodion. A wire splint placed at proper angle was now adjusted, and the patient placed in the college hospital, where he constantly and steadily improved, until now he is very nearly well.

*S. W., aged* 31. Neuralgia in the popliteal space; the peroneal nerve being the one affected. Has been under constant treatment for fourteen months, but has no relief. An incision was made, and a small portion of the nerve was removed. This gave almost immediate relief, and the patient has been removed cured.

*D. K., aged* 3 *months*. Congenital club foot. Heels drawn up very high, and both feet turned in. Chloroform administered and the tendo achillis divided three-fourths of an inch above the os calcis. The foot was then forcibly extended. Not a drop of blood flowed, and the patient was discharged. One week afterwards she was brought to clinic, and the feet were in excellent position, and no untoward symptom had followed the operation.

*P. W., aged* 22. Last April, four years ago, he was ill for three weeks with an attack of pleurisy. At that time pus formed and opened externally below the scapula, about four inches in front of the spinal column, which, by ulcerative action, has extended outward and downward until now he has a sinus on the left side of the chest nine inches in length, and between the sixth and seventh rib, communicates with the cavity of the chest. A silver tube was introduced and pus drained off. Directed that the wound should be daily washed with a solution of permanganate of potash. Necrosis of the fifth and part of the sixth ribs. The fifth rib was removed entirely on the left side, and the sixth rib was scraped. The left lung was wholly collapsed.

